# New products

**DOI:** 10.1155/S1463924688000227

**Published:** 1988


Journal of Automatic Chemistry, Vol. 10, No. 2 (April–June 1988), pp. 111–119

### New products

### Gas chromatograph

The 8700 GC offers simultaneous dual channel operation with a full colour VDU for enhanced user interaction. Powerful integral data processing, with multi-level calibration and multiple reference peak facilities, easily handles the most complex chromatography, making the instrument ideal for research environments as well as routine analysis.

Features include:

- Single or simultaneous dual channel chromatographic operation.
- Full colour VDU with softkey user interaction.
- Dual-channel real-time or post run graphics, split screen or overlaid.
- Simple graphics manipulation for comparison of sample and reference chromatograms.
- Enhanced software for independent integration of both channels, multi-level calibration, and multiple reference peaks, with re-integration facilities.
- Computer communications for transfer of GC methods, raw and re-integrated data and reports.
- Fully automated, analytical sequencing software is included as standard for routine applications.

*For further information contact Perkin-Elmer Ltd, Post Office Lane, Beaconsfield, Buckinghamshire HP9 1QA, UK.*

### Centrifuge brochure

Beckman's range of GP series centrifuges is described in an illustrated brochure. There is a choice of models that fit under, as well as on top of, the laboratory bench, and the GP centrifuges offer a 3-litre capacity, 3750 rpm/3200 *g* with horizontal rotor or 6400 rpm/5642 *g* with fixed angle versions, automatic soft-start and two-stage disc brake for rapid but gentle acceleration.

Modular disc adapters are available for every popular sized tube and the GP is the first compact centrifuge able to run 250 ml conical-tip tissue culture bottles – up to four per run in special sleeves. It also spins 750 ml, 500 ml and general 250 ml bottles and accommodates up to eight 96-well microplates as well as blood bag cups for single, double, triple and quad packs and aerosol canisters, with bucket covers to contain broken tubes. Each model is available with or without refrigeration.

*Further information from Beckman Ltd, Progress Road, Sands Industrial Estate, High Wycombe, Buckinghamshire, UK. Tel.: 0494 41181.*

### Dual target sample introduction probe

Fast atom bombardment mass spectrometry (FAB-MS) is an indispensable technique for the analysis of thermolabile, non-volatile compounds and polar compounds. Accurate mass measurement by conventional FAB-MS has been made by mixing the internal reference sample, sample and matrix materials at a suitable ratio and placing the mixture on a single sample target of the FAB probe. However, as the ionization efficiency of the sample cannot be known in advance, the adjustment of the optimum concentration ratio between the sample and internal reference sample must be made by trial and error.

A new dual target FAB probe has been developed by Jeol Ltd to overcome such problems.

The probe tip has two faces which are loaded with two different samples, which can be measured by rotating the probe by 180°. One face is loaded with an unknown sample and the other with a reference sample, and the target face is changed over alternately at each scan. This probe can measure inorganic salts such as caesium iodide as the reference sample, and permits mass measurement in a high mass range above m/z 1500, which has not been possible by the conventional method.

The new probe is described in two application papers available from Jeol. Work detailed includes mass measurements on compounds with molecular weights of 362·2 to 1857·9. These include angiotensin-1 and gamma-endorphin, hydrocortisone, erythromycin, alpha-cyclodextrin, bradykinine, bracitracin A and alpha-MSH.

*Details of the new probe and copies of the papers are available from Jeol Ltd, Jeol House, Grove Park, Colindale, London NW9 0JN, UK. Tel.: 01 205 6376.*

### Sample scheduler software

It is a routine requirement for any analytical laboratory concerned with production control, to have a prescribed schedule of sampling times, dates and locations. Similarly, health control and water control laboratories need to know when and where to collect samples. Perkin-Elmer has now released software, the sample scheduler package, to fully automate this aspect of laboratory information management. The sample scheduler package is an enhancement package to Perkin-Elmer's laboratory information management system (LIMS 2000).

The major features of the sample sheduler package are:

- A spreadsheet format diary: allowing a laboratory to specify, up to a year in advance, when a sample should be taken from which sampling point and which tests are to be performed at the sampling point or in the laboratory. Sampling points could be river stations, soil areas or, on completion of certain reactions, within a multi-stage chemical process.

- Collection lists: indicating when and from where the samples are due for collection. These can be produced at any time, again up to a year in advance if necessary, and with optional labels to attach to samples at time of collection.

- Sample receipt: an autoscheduling feature works overnight to search the computer data-base for all the samples expected the next day and creates a current file for them. This saves the analyst time when logging samples on to the system. The software can cope with samples that have been lost or will be sampled at a later date out of sequence.

From scheduling sample collection to report generation, the LIMS 2000 family provides a total solution to information management problems.

*For further information contact Perkin-Elmer Ltd, Post Office Lane, Beaconsfield, Buckinghamshire HP9 1QA, UK.*

### Sample manipulation

Applied Research Laboratories (ARL) has announced two systems for the automatic manipulation of samples. The SMS-100 and SMS-200 are standard extensions of the ARL 3460 optical emission spectrometer and both provide a fully automatic instrument operation eliminating the need for operators to handle samples.

The SMS systems provide the following automatic facilities:

- Sample preparation.
- Sample transport to the spectrometer spark stand.
- Sample analysis.
- Sample filing following analysis.
- Cleaning of electrode and spark stand table.
- Instrument standardization (option).
- Check of instrument operation using control samples (option).

The more sophisticated version, the SMS-200, employs one of the most advanced industrial robots on the market. The smaller SMS-100 version is equipped with a proven and widely accepted industrial robot capable of handling up to 40 sample analyses per hour. Each robot possesses an armlike frame and stands on a subframe which is linked electrically and mechanically to the spectrometer.

The spectrometer equipped with an SMS-100 or SMS-200 system can be used in various environments. For example:

- In close proximity to the production process, installed in a cabin or shelter (local laboratory version).
- In a central laboratory, remote from the production process. In this case, the user needs to install a long distance automatic sample transport and preparation system to achieve complete automation (central laboratory version).

In fully automatic operation, the SMS-200 can handle as many as 60 sample analyses per hour; far more than any operator could sustain over a working shift.

Apart from the savings in manpower which can be achieved by employing the SMS-100 or SMS-200 sample manipulation systems, further reductions in production costs are obtained because the systems:

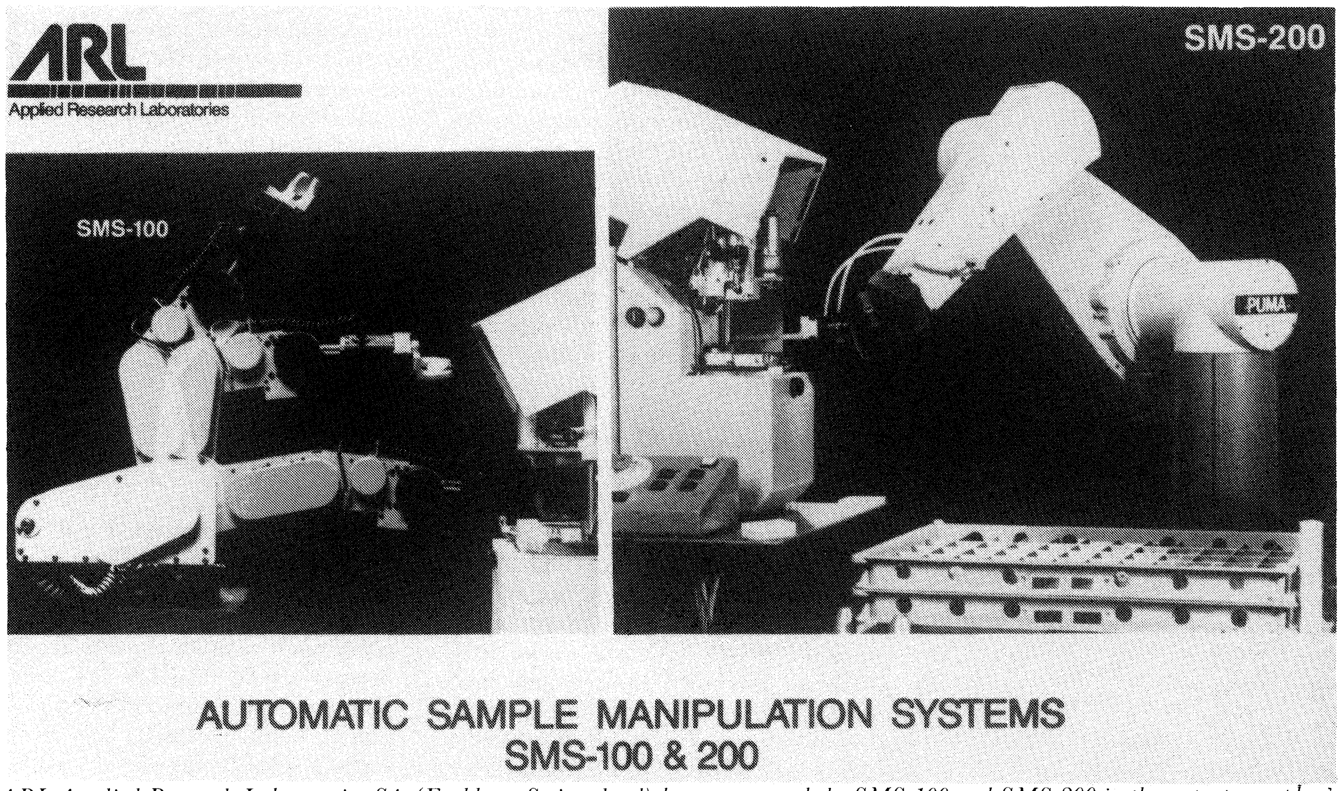

*ARL Applied Research Laboratories SA (Ecublens, Switzerland) have announced the SMS-100 and SMS-200 in their spectrographer's newspaper 'ARL News'.*

- Reduce the response time between sampling and availability of the sample analysis.
- Increase the sample analysis cadence.
- Increase overall reliability by eliminating possible human error.

All result in an increased return on the spectrometer investment, improved laboratory productivity and short payback period.

*Details from ARL Applied Research Laboratories SA, En Vallaire, CH 1024 Ecublens, Switzerland. Tel.: 021 34 97 01.*

### Quality control

The quality control laboratory is the key element in ensuring that products conform to specifications, internal limits and possible legislative requirements. QCLIMS is a laboratory information management system designed by Perkin-Elmer to assist specifically in maintaining product quality in the pharmaceutical, cosmetic and food and drink industries.

It has been successfully installed at sites in the UK, Europe and North America, feedback of ideas from the customer base has assisted Perkin-Elmer in the development of QCLIMS revision 1.1.

QCLIMS revision 1.1 is an enhanced version of this popular software package. It contains substantially increased functionality and provides the user with a well structured and sound product which has been engineered in line with good laboratory practices. This means that laboratory personnel can maintain a great degree of confidence in their analytical results as well as in the decisions derived from such data.

The core of the system is the QCLIMS database, which is divided into a number of data sets. These contain details of samples, tests, specifications and other information necessary to run the system. The configuration of the database is flexible and data sets can be defined to meet the needs of laboratory and production personnel.

The 'applications' software is of modular design and performs operations from specification management and sample log-in to results entry and certificate of analysis production. QCLIMS provides the means to effectively control information generated in the laboratory and so while data is organized, skilled laboratory analysts are freed to analyse the samples.

QCLIMS revision 1.1 features enhancements to most of the application modules and operates with the LIMS 2000 software on a wide range of Perkin-Elmer 32-bit minicomputers. Any suitably configured IBM PC or compatible can also be incorporated into the QCLIMS system to work as a text and graphics terminal and provide a system which has the benefits of both a centralized database and distributed processing.

*Further details from Perkin-Elmer Ltd, Post Office Lane, Beaconsfield, Buckinghamshire HP9 1QA, UK. Tel.: 04946 6161.*

### Fluorescence detectors

Severn Analytical, the UK's largest independent supplier of HPLC and SFC instrumentation have introduced a new range of fluorescence detectors – the FD series.

All competitively priced, the instruments greatly enhance Severn's capability as a specialist supplier of detectors for HPLC.

The three instruments in the range all feature a powerful 10 W xenon flash source and are designed for the fast and convenient interchange of flow cells and cuvettes for microbore, analytical and preparative applications. Measurements of non-flowing samples, such as those from fraction collectors, can be taken with a cuvette accessory.

The FD-100 is an advanced filter fluorimeter with a wide selection of the highest quality excitation and emission filters which can be interchanged in seconds.

The FD-200 features the combination of a high efficiency monochromator for excitation and emission filters common to the FD-100.

The top-of-the-range FD-300 is a dual monochromator instrument, with continuously variable excitation and emission wavelengths. Incorporating high throughput monochromators and offering low stray light, it is an ideal detector for applications where the excitation and emission wavelengths are closely separated.

The detectors will find applications in many areas, including pharmaceutical analysis, food analysis, clinical assays, organic synthesis and polymer/plastics analyses.

*For further technical information contact Dave Green, Severn Analytical Limited, Unit 2b, St Francis' Way, Shefford Industrial Park, Shefford, Bedfordshire SG17 5DZ, UK. Tel.: 0462 816406.*

### Titrator

With a built-in data-base of 15 standard methods, the new Mettler DL25 titrator is suitable for both routine work and for development. This titrator has significant advantages, particularly for analytical laboratories that require precise, clear, results quickly.

The 15 standard methods held in the data-base can be used for acid, base and redox titrations, non-aqueous or photometric titrations, and also for the determination of chlorides. The DL25 titrates automatically to two endpoints or to equivalent points; it determines p- and m- values, pH values, TAN/TBN values (total acid and total base numbers) according to ASTM/DIN, calculates half-neutralization value (HNV) and carries out pH stat and back titrations.

In all cases where the standard methods, although optimized in many practical trials, do not exactly meet the user's requirements, the method can be adapted to the particular application with configuration parameters entered in a dialogue with the DL25. These standard methods can then be stored in a special memory which will hold up to 50 methods. A code prevents the method parameters from being altered accidentally.

Mettler balances can be connected for transferring sample weights automatically. A Mettler GA44 printer or a commercial dot matrix graphics printer then logs the titration data in detail. The Mettler ST20 sample changer (optional) or robots or computers can also be connected.

*More information from Mettler Instrumente AG, CH 8606 Greifensee, Switzerland.*

### Binary gradient controller

The Aston LC controller is a binary gradient controller, which is compatible with most stand-alone LC pumps. Gradient profiles and timed events schedule are created on the graphics screen of an IBM or compatible PC. Methods can be stored on disk and then downloaded to the controller where its battery backed RAM storage means that they are always available for use.

Gradient programs are powerful, allowing up to 10 segments, each of which can have a different profile and flow rate. The controller integrates well with automated systems and provides appropriate handshaking signals for other equipment.

With a method in the controller's memory, the PC may be disconnected, an LCD and status lights on the controller providing information to the chromatographer on the method, status and chromatographic conditions.

Outputs are available on the controller to drive most modern frequency, analogue or RS232 driven pumps and now it is possible to assemble a gradient HPLC system using two pumps from different manufacturers.

The Aston LC controller requires a PC or compatible with 512K RAM and CGA, EGA or Hercules graphics, and an RS232 interface. As a limited period introductory promotion Aston Scientific will be shipping the controller with a free Amstrad PPC640 portable PC.

*For further information contact Martin Perry, Aston Scientific Ltd, 49 Long Plough, Aston Clinton, Buckinghamshire HP22 5HD, UK. Tel.: 0296 630304.*

### Revision to Lab Manager

CALS Lab Manager Revision 6.0, an integrated laboratory information management system (LIMS), is now available from Beckman. Revision 6.0 is the current release version of Lab Manager software for DEC VAX and H-P computers. It contains many enhancements and much new functionality suggested by the large, active CALS User Group.

Revision 6.0 provides users with total full screen data entry and execution of most functions. Full screen operation can be described as a 'fill in the blanks' approach to a computer interface, similar to entering data on a blank form in a laboratory notebook. This capacity makes Lab Manager Revision 6.0 simple to use, thus reducing the personnel training burden.

In previous Lab Manager software revisions, functions for sample log-in, testing and validation were performed by the full screen mode of operation. Revision 6.0 extends this capability to approval, entry of retests and dictionary maintenance. Test definitions, product specifications, calculations and test scheduling may be maintained and updated on-line. Review and approval of all such changes may be required before putting them into use. All previous test specifications, product specifications and other dictionary entries can

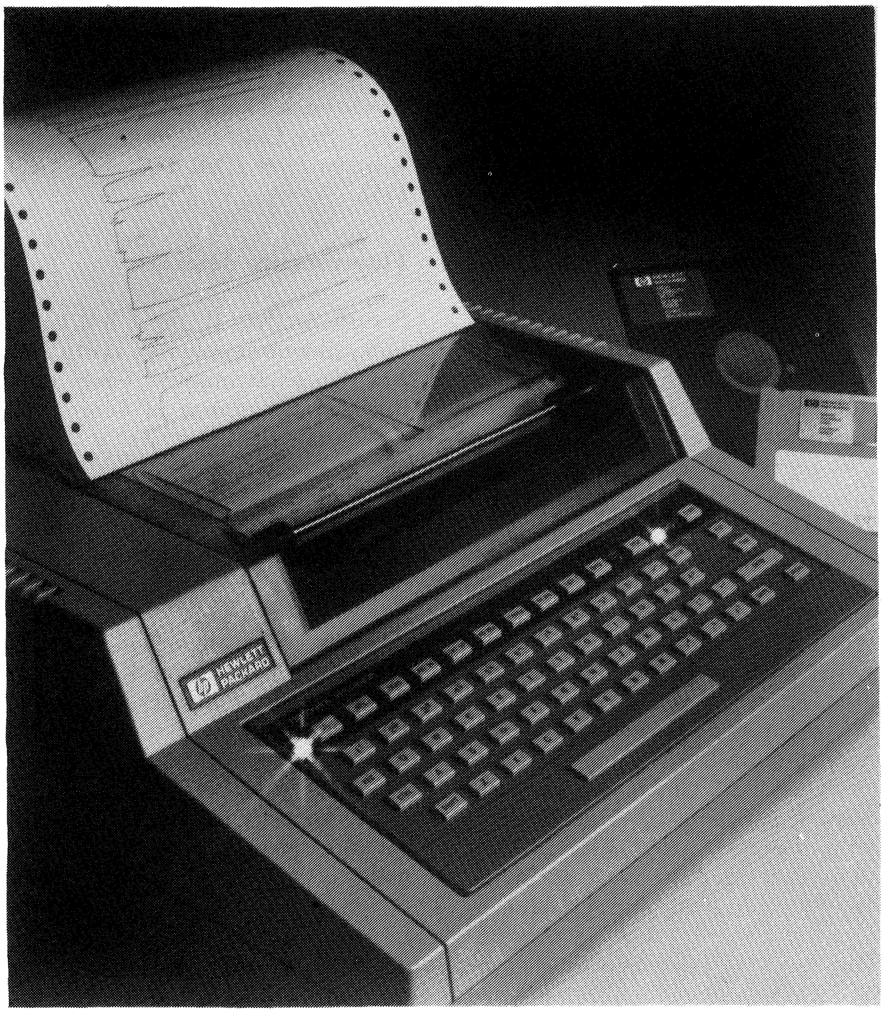

*The new chromatography integrator from Hewlett-Packard (HP 3396A), which offers a full range of functions for less than £1500. For this price, it provides wide-ranging capabilities including storage and re-plotting of chromatograms, negative peak handling, ESTD% reporting, multi-level calibration, method storage and automation. A single integrated circuit board contains all system electronics, and the integrator can be used to control and implement a complete analysis, and to generate and annotate reports. Full information from the Analytical Instrumentation Group, Hewlett-Packard Ltd, Miller House, The Ring, Bracknell, Berkshire RG12 1XN, UK.*

be maintained on-line for review where laboratories are subject to compliance with government regulations. All changes are recorded – who made the change and the time and date.

Lab Manager Revision 6.0 also improves data output. Users may scroll or pan the screen horizontally. Report commands are now permitted with full screen data entry. Lab Manager Revision 6.0 offers the ability to add AUTOLOG, an optional software package for automatic sample log-in; it is especially useful for applications where a multitude of samples are generated on a periodically scheduled basis, such as chemical processing plants.

*For additional information, specifications or a demonstration, contact: John Boother, Laboratory Automation Operations, Beckman RIIC Ltd, Progress Road, Sands Industrial Estate, High Wycombe, Buckinghamshire HP12 4JL, UK. Tel.: 0494 41181.*

### Metrohm and Howe

From 1 January Metrohm equipment will be sold in the UK by V. A. Howe & Company Ltd. A catalogue describing Metrohm's products is now available from Howe. The range includes:

- Dispensers and diluters.
- 'Titroprocessors'.
- Karl Fischer titrators.
- Ion chromatographs.
- Photometers.
- Conductometers.
- pH, redox and ion meters.

Howe are backing up the dealership with seminars, service contracts, applications advise and a users' club.

*Copies of the catalogue from V. A. Howe & Co. Ltd, 12–14 St Ann's Crescent, London SW18 2LS. Tel.: 01 874 0422.*

### Atomic absorption spectrophotometer

The model 5100 is a fully automated multi-element system for any AA application and is optimized for flame, furnace and mercury/hydride sampling. It will readily perform both conventional flame and furnace AA, as well as Zeeman-corrected graphite furnace analysis.

The Model 5100 PC has been designed to be easy to use. The colour software works within a GEM environment and control of the instrument is via a mouse pointer and function keys. Drop-down menus, and multiple windows providing between one and five simultaneous 'views' into the system, offer maximum versatility with peak performance.

The 5100 PC system uses an IBM of fully compatible PC and includes a full complement of options and accessories to expand system capability as analytical needs change.

*For further information, contact Perkin-Elmer Ltd, Post Office Lane, Beaconsfield, Buckinghamshire HP9 1QA, UK. Tel.: 04946 6161.*

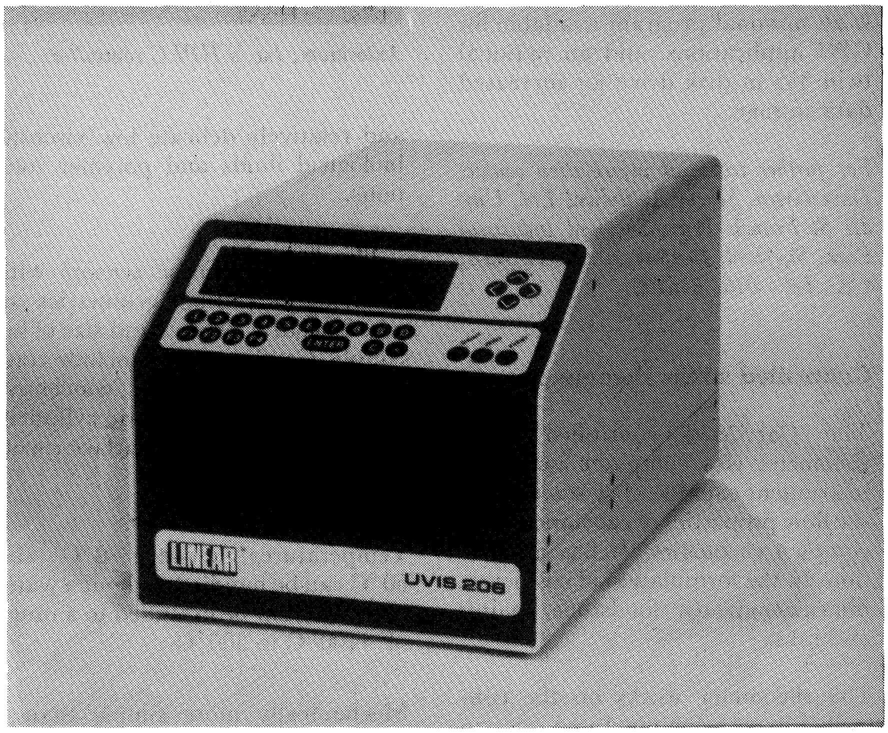

*Linear Instruments Corporation has introduced its high speed UV-Vis HPLC detector, Model 206, which is designed to out-perform the more expensive Photodiode Array (PDA) HPLC detectors. The Model 206 can monitor 32 different wavelengths simultaneously during the running of a chromatogram, and can provide chromatogram tracings at four selected wavelengths, including on the fly spectra.*

*Using a high speed grating drive mechanism, the detector can achieve sensitivity levels above those achieved by PDA units.*

*The Model 206 is entirely user programmable in simple, easy to understand language.*

*The Model 206 uses Linear's complete line of externally mounted flow cells, including: capillary, microbore, analytical, semi-preparative and variable pathlength preparative cell.*

*The operating range of the instument is 190–800 nm, and it is priced well under $10 000.*

*For complete information, contact Linear Instruments Corporation, 500 Edison Way, Reno, Nevada 89502, USA. Tel.: 702 786 3636.*

### Integration for chromatography

Severn Analytical have introduced the Chromatocorder 12, a low-cost single channel integrator for chromatography. The integrator is compatible with any GC, LC, SFC or TLC. Although compact it incorporates an integral full-width printer/plotter. Remote control and long-term power fail memory back up are also included as standard.

Key features of the instrument are the extensive 'Help' messages designed to improve ease-of-use, and chromatogram storage and replay with base-line construction and naming of peaks.

To further expand its capability there is an optional program available for GPC applications, and an optional twin 3½ in disk drive for increased data storage.

*For further technical information contact Dave Green, Severn Analytical Ltd, Unit 2B St Francis Way, Shefford Industrial Park, Shefford, Bedfordshire SG17 5DZ, UK. Tel.: 0462 816406.*

### Controlled stress rheometer

The CarriMed controlled stress rheometer is a computer controlled instrument capable of characterizing the flow properties of substances with complex rheologies. It has applications in the formulation of cosmetics, pharmaceuticals, foodstuffs, paints and inks.

The rheometer works on the controlled stress principle. In addition to standard measurements that can be made on a rotational controlled-shear rate instrument, it is suited to studies on materials at rest, interactions between phases and molecular interactions. Moreover, stress controlled situations, such as the settling of suspensions, film levelling, and flow under gravity, can be simulated.

Operating modes include creep and oscillation tests, plus controlled stress and controlled shear flow modes. The instrument can handle a wide range of samples, including visco-elastic inks, thixotropic paints, fibrous and heterogeneous foodstuffs,

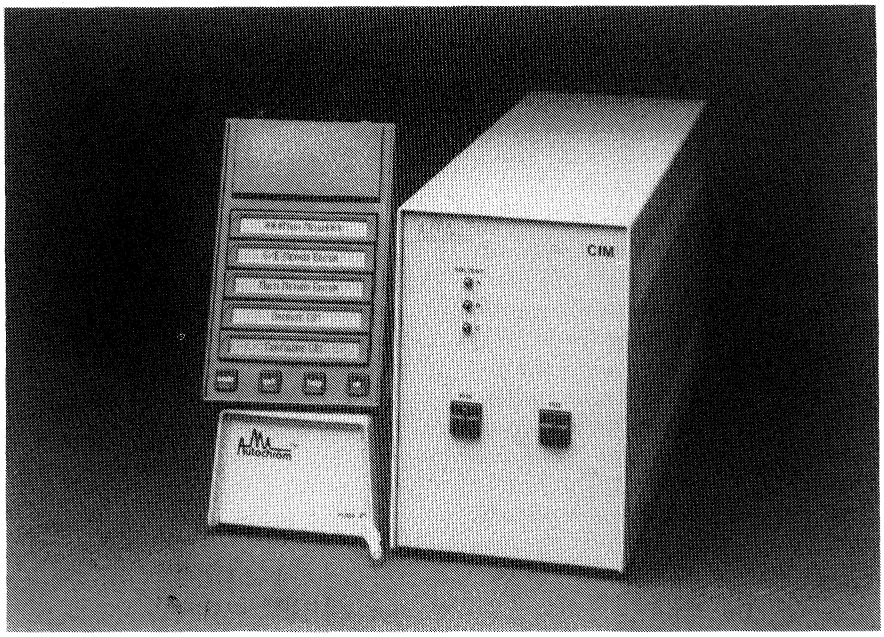

*Autochrom, Inc.'s HPLC controller.*

and relatively delicate low viscosity biological fluids and polymer solutions.

The user can choose sensors with different measuring geometries to suit the characteristics and size of his sample. Configurations include cone and plate, parallel plates, concentric and double-gap concentric cylinders and special designs for surface rheology and fibre testing.

Temperatures between 8 °C and 60 °C can be handled without a water bath; this can be extended to a range of −100 °C to 300 °C.

Mechanically more simple than a rotational rheometer, the same controlled stress system can be applied to many different substances simply by software changes. Experimental variables can be stored in the computer and set for a given experiment. Once set-up, the full rheological characterization is handled entirely by the computer.

The controlled stress rheometer can characterize the flow behaviour of both high and low viscosity materials from $2{\cdot}5 \times 10^{-2}$ mPasec to $2{\cdot}5 \times 10^{9}$ mPasec. Shear rates can be measured from $5 \times 10^{9}$ to readings of $10^{-5}$ $sec^{-1}$ and below, depending upon the geometry and technique applied.

*Further details are available from Mrs Sue Chick, Carri-Med Ltd, Glebelands Centre, Vincent Lane, Dorking, Surrey, UK. Tel.: 0306 886180. The US distributor is MiTech Corporation.*

### HPLC gradient/system controller

The Autochrom Model 300 family of HPLC controllers upgrade existing isocratic HPLC systems. The Model 300 family of controllers blend up to three solvents using one pump or two solvents using two pumps. Proprietary proportioning algorithms (AAPT) can upgrade pumps to provide gradient accuracy and repeatability comparable to integrated solvent delivery systems costing two to three times more. Four curve types, battery-backed memory, and six external events expand the versatility. Configurations for either stand-alone or computer-based HPLC systems are available.

*Details from Autochrom, Inc., PO Box 207, Milford, Massachusetts 01757, USA. Tel.: 617 478 2670.*

### Enzyme-linked HPLC

The combination of an enzyme-linked assay and HPLC is a simple routine method to analyse acetylcholine and choline in the p mol range. These are separated by HPLC and then passed through the enzyme-reactor, where hydrolysis of acetylcholine, oxidation of choline and formation of $H_2O_2$ take place. The $H_2O_2$ is then measured by the electrochemical detector. Complicated extraction and concentration procedures (for instance for the analysis of tissue homogenates) are unnecessary. There is no loss in enzyme activity for several months in continuous mode action.

*More information from Biometra, Biomedizinische Analytik GmbH, Wagenstieg 5, D 3400 Göttingen, FR Germany.*

### Amino-acid analysis

Beckman's recently introduced System Gold Personal Chromatograph is now available in a special configuration for amino-acid analysis. Ion exchange chromatography can be used with this system and there is a choice of ion exchange columns for separating either physiological fluids or hydrolysates.

For results that are unaffected by sample matrix or derivative stability during separation, the System Gold chromatograph uses cation exchange with ninhydrin post-column derivitization and detection at 500 nm. This method separates and detects all amino-acids in a single process.

The system can also be easily configured for high-sensitivity binary gradient analysis of PTH, PTC and OPA derivatives using the company's Ultrasphere columns.

The user controls the System Gold personal chromatograph for amino-acid analysis with an IBM PC-XT, PC-AT or PS 2 model computer, and views chromatograms, integration data and system status on the monochrome or colour screen. The system's autosampler handles up to 80 samples with injection volumes from 1 to 250 μl programmable in 1 μl increments. The UV/VIS detector is fully programmable and includes a tungsten source and a post column reactor are also included in the system.

*Details from Beckman Ltd, Progress Road, Sands Industrial Estate, High Wycombe, Buckinghamshire, UK. Tel.: 0494 41181.*

### Atomic spectrometers

A new four page colour mini-catalogue for atomic absorption spectrophotometers, sequential and simultaneous ICAP spectrometers, arc/spark direct reading emission spectrometers, and solid sampling plasma spectrometers was published in January by Thermo Jarrell Ash, Franklin, MA. The catalogue is intended to provide spectroscopic information to analytical laboratories performing elemental analyses – ranging from the analyses of metal castings, to the analyses of a few dozen solution samples a week for one or two trace metals, to the routine determination of large numbers of elements in hundreds of samples a day.

*Copies from Thermo Jarrell Ash, 8E Forge Parkway, Franklin, Massachusetts 02038, USA. Tel.: 617 520 1880.*

### Applications bibliography

Over 500 articles and procedures, which cite the use of Analytichem solid phase extraction products in the extraction and purification of a wide variety of chemical compounds found in biomedical, clinical, environmental, food and general industrial applications, are listed in Analytichem's applications biography published in January 1988.

Each entry lists the complete reference, as well as the compounds of interest, the matrix from which the isolates were extracted, the method of final analysis and the type of Analytichem sorbent used.

The company's Bond Elut, Bond Elut LRC and Chem Elut and Tox Elut extraction columns can solve time-consuming sample preparation problems by extracting, purifying and concentrating compounds of interest in minutes instead of hours. The bibliography highlights analytical methods using solid phase extraction in conjunction with a variety of analytical techniques. These include HPLC, GC, GC/MS, UV spectroscopy, ion selective electrode analysis, RIA, enzyme immunoassay NMR, LC/MS and TLC.

*More information from Analytichem International, PO Box 234, Cambridge CB2 1PE, UK. Tel.: 0223 328177.*

### pH/blood gas analysers

An improved family of pH/blood gas analysers from Instrumentation Laboratory (IL) offers a choice of sampling modes that now includes sample injectioin, as well as aspiration from all common containers. In addition a 'Help key' has been added to simplify operation and troubleshooting.

The 'Help key' is now available on IL's mid-priced *System 1306* analyser. The key provides instant access to some 40 different 'help' messages. As well as diagnosing any problem, it provides directions on the various operating modes, such as explaining how to turn certain parameters on or off. It even knows if the unit is interfaced with an IL *CO-Oximeter*. The 'Help key' feature is available in five languages.

*A brochure describing the pH/blood gas analysers is available from Instrumentation Laboratory, 113 Hartwell Avenue, Lexington, Massachusetts 02173, USA. Tel.: 617 861 0710, or from Instrumentation Laboratory SpA, Milan, Italy. Tel: 39 2 25221.*

### Chiramonitor

Recent developments in the monitoring of optically active compounds has shown the need for a universal method of quantitatively detecting chiral enantiomers of drugs and other pharmaceutical formulations. Enantiomers can show therapeutic properties in one isomer, whilst the other is toxic.

The Chiramonitor, manufactured by Applied Chromatography Systems, will detect any compounds showing optical activity. The detector is sensitive down to ngm levels and linear over wide concentration levels.

A laser source, coupled with a photodiode detection system, allows accurate and reproducible HPLC analysis.

By using enantiomer ratioing procedures, it is possible to analyse the exact concentrations of enantiomers, without the requirement for separation. Thus, expensive and specific chiral columns are not necessary in some applications.

*Details from Applied Chromatography Systems, Heapy Street, Macclesfield, Cheshire SK11 7JB, UK. Tel.: 0625 34575.*

### IL Test reagents

New IL Test reagents for CK/CPK, uric acid, cholesterol and ALT/SGPT have been introduced by Instrumentation Laboratory (IL) for the Monarch chemistry system

The new reagents offer increased on-board stability, improved linearity and faster assay time, resulting in more tests per kit, lower cost per test and faster turn-around of test results.

The new IL Test for CK/CPK is an NAC activated procedure with an expected range of 14–156 U/l at 30 °C. It has a reconstituted life of seven days at 2–6 °C and is linear to 1800 U/l. Assay time has been reduced to less than 5 min.

The new IL Test for uric acid is a single reagent procedure with reconstituted stability of two months at 2–6 °C and linearity to 20 mg/dl.

The new IL Test for cholesterol uses a concentrated liquid reagent with open container stability for 30 days at 2–25 °C. This 4-min assay requires only 50 µl of reagent per test.

The IL Test reagent for ALT/SGPT has a reconstituted stability of 14 days at 2–15 °C.

The new IL Test reagents are packaged in easy-to-use, prefilled BoatIL containers which are loaded directly into the Monarch Chemistry System. Containers are bar-coded so the system can identify them automatically.

*For a detailed brochure, contact Instrumentation Laboratory, Lexington, MA 02173, USA. Tel: 617 861-0710. Tlx: 92-3440 (247084 ILLX UR, via RCA); or Instrumentation Laboratory SpA, Milan, Italy. Tel: 39-2-25221. Tlx: 330112 ILSPA I.*

### Compact printer

Another addition to the wide range of products introduced during the last 12 months is now available from Salter A & D. Weighing 330 g and operating on either a.c. or battery power, the unit has been specially built for use with a wide range of weighing indicators and electronic and counting balances.

Standard features include: a serial thermal dot matrix printer; a full range of statistical functions showing weight and counting data in a number of operations with the added ability to calculate up to 999 data blocks. The AD-8117 is priced at £399.00.

*Details from Salter A & D, Spring Road, Smethwick, Warley, West Midlands, UK. Tel.: 021 553 1855.*

### TLC Autospotter

A comprehensive range of TLC hardware is now available from Whatman. The range includes separating chambers, dipping chambers, UVis systems and features the Autospotter TLC sample application system.

The Autospotter is designed specifically for routine quality-control applications and allows the simultaneous application and concentration of up to 20 samples on 20 × 20 cm or 20 × 10 cm TLC plates. The sample volume can be varied between 5–50 µl. Spot diameters, after evaporation by the built-in drying system, are held between 1 mm and 5 mm.

The Autospotter is ideal for use with Whatman Linear-K, pre-channelled and conventional TLC plates.

*More information from Whatman Ltd, Springfield Hill, Maidstone, Kent ME14 2LE, UK. Tel.: 0622 61681.*

### Chromacol bench-top crimper

Crimpmate (see p. 119) was built to ease the work-loads met by growing numbers of laboratories and other work units with increasing vial crimping requirements.

The main body of the system is supported by a stem on a solid base. It can be raised or lowered to suit the personal needs of the user by means of a simple finger knob. Because of the unique leverage system, only about a quarter of the normal muscle pressure is used.

The jaws fit into a holder in the main body in such a way that they can be interchanged in less than 8 seconds. Each jaw head has a fine tuning knob which allows a given jaw head to be adjusted to deal with variations in seal thickness and in vial collar tolerances. Four jaw sizes are presently available: 8 mm, 11 mm, 13 mm, 20 mm.

*Details from Chromacol Ltd, Glen Ross House, Summers Row, London N12 0LD. Tel.: 01 368 7666.*

### Bioscreen

The first of a new generation of Bioscreens has been purchased by Unilever Research and installed at their Colworth House laboratory in Bedfordshire, UK. This latest version of Labsystems' microbiological growth analyser, known as the Bioscreen-C, contains an incubator which is continuously adjustable from 15°C to 55°C.

The instrument purchased by Unilever is to be used in a research project being run jointly by Drs Martin Cole and Martin Jones of the microbiology department at Colworth. The Bioscreen will be utilized for predictive modelling of the bacterial flora associated with a variety of foodstuffs.

Bioscreen-C is available with a number of different sofware programs dedicated to routine food quality control, research on anti-microbial compounds, bioassays and toxicity testing. As well as aerobic and anaerobic bacteria, Bioscreen may be used for monitoring yeasts and moulds.

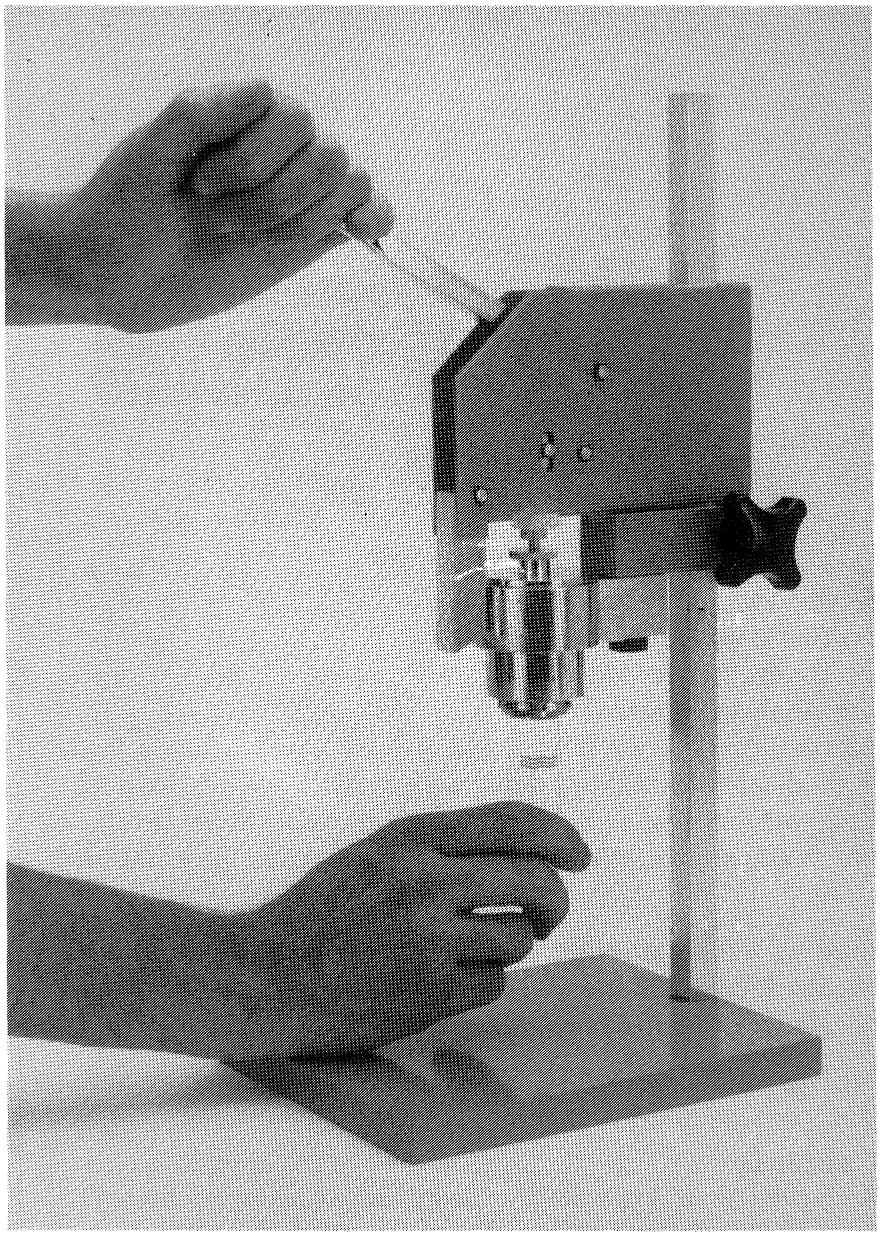

Chromacol Ltd's Crimplate 'easy action' bench-top crimper. (See p. 118)

*More information from Labsystems (UK) Ltd, 12 Redford Way, Uxbridge, Middlesex UB8 1SZ, UK. Tel.: 0895 38421.*

### Chemical word-processor

Chemical word-processing is possible on Hewlett-Packard's Vectra personal computer – the user can create documents incorporating graphical output from data collected and processed by the HP ChemStation.

The ChemText package, which was developed by Molecular Design Limited (California), is designed to provide an accurate, reliable and effective means of communicating chemical information. Reports, documents and scientific papers may be created easily on the HP Vectra or equivalent personal computer, and structures drawn directly on screen using the mouse.

A word-processor that handles chemical symbols, molecular structures, mathematical formulae and other special characters, ChemText outputs to the printer what is seen on screen. Text can be produced incorporating chemical structures, reaction diagrams, equations and flowcharts. The software supports a wide range of printers, including the HP LaserJet and HP ThinkJet.

ChemText allows information from a variety of sources, such as the HP ChemStation, to be integrated into a single document. Images imported to ChemText remain computable – the chemist can, for example, move a molecular diagram to and from ChemText documents, data-bases, modelling programs etc. Graphs, chromatograms and other diagrams may be manipulated and labelled to produce a complete presentation quality document.

*Details from Wendy Holt, Hewlett-Packard Ltd, Miller House, The Ring, Bracknell, Berkshire RG12 1XN, UK. Tel: 0344 424898.*

### New family coagulation analysers

A new family of three *ACL*™ automated coagulation analysers has been announced by Instrumentation Laboratory (IL). *The ACL 100* is designed for routine clinical analysis in laboratories running about 30 tests per day. It automates all routine clot-timing analyses, including simultaneous analysis of both PT and fibrinogen. With 1100 data points averaged into each test result, the system offers superior accuracy and precision. Typical analytical precision within a run is ≤1% coefficient of variation for PT when using IL reagents with fresh plasma.

*The ACL 200* performs chromogenic substrate assays, in addition to all routine clot-timing analyses.

*The ACL 300* provides all the capabilities of the *ACL* 200 system plus higher throughput, a built-in thermal printer and an additional RS232C port. Recommended for larger hospitals and research laboratories, it is designed to interface with an external PC computer. A built-in rotor preheater increases analytical throughput from 110 to 175 tests per hour for PT and from 80 to 115 tests per hour for APTT. An optional Research Program allows the user to vary test conditions and display formats.

